# Stable Management

**Published:** 1883-01

**Authors:** 


STABLE MANAGEMENT AND THE PREVENTION OF DISEASE AMONG HORSES IN INDIA. By J. I. Meyrick, F.R.C.V.S.

This little book, of less than one hundred pages, contains a large amount of information interesting to veterinarians and exceedingly useful to those who intend practising in tropical countries. The author, believing that "prevention is better than cure," devotes one-half his book to the consideration of hygienic and preventive measures in the care of horses. Diseases and their treatment, however, are not neglected, and the article, "Anthrax," contains all of importance that can be said of that terrible disease. There will be found also some practical hints as to the care of camels, oxen and other animals.

